# Lupus serum IgG induces microglia activation through Fc fragment dependent way and modulated by B-cell activating factor

**DOI:** 10.1186/s12967-019-02175-0

**Published:** 2019-12-21

**Authors:** Chunshu Yang, Xiaoyu Hou, Qianhui Feng, Yingzhuo Li, Xuejiao Wang, Ling Qin, Pingting Yang

**Affiliations:** 1 Department of 1st Cancer Institute, First Affiliated Hospital, China Medical University, Shenyang, 110001 People’s Republic of China; 2 Department of Rheumatology and Immunology, First Affiliated Hospital, China Medical University, Shenyang, 110001 People’s Republic of China; 3 Department of Physiology, School of Life Science, China Medical University, Shenyang, 110122 People’s Republic of China

**Keywords:** FcγR, BAFF, Systemic lupus erythematosus, Immunoglobulin, Neuroinflammation

## Abstract

**Background:**

Neuropsychiatric manifestations are frequent in patients with systemic lupus erythematosus (SLE), yet the etiology and pathogenesis of brain damage in SLE remains unclear. Because the production of autoantibodies, formation and deposition of immunocomplexes are major serological characteristics of SLE, the elevated level of serum immunoglobulin may contribute to brain tissue injury of SLE. To testify this, in this study, we examined whether immunoglobulin G (IgG) in the serum of SLE patients affects the cellular functions in central nervous system and the potential mechanism.

**Methods:**

In vivo intracerebral injection of SLE-serum in mouse was used to activate microglia and the production of pro-inflammatory cytokine was assessed by ELISA. Sera was divided into IgG and IgG depleted fractions, while IgG was further divided into Fc and Fab fragments to examine which part has an effect on microglia. Flow cytometry, immunofluorescence and quantitative PCR (qPCR) were used to verify the synergistic effect of B-cell activating factor (BAFF) on IgG stimulation of microglia.

**Results:**

We found that IgG in lupus sera can induce M1 activation of brain microglia following intraventricular injection into normal mice, and BAFF facilitates this process. In vitro, we identified that IgG bound to microglia through Fc rather than Fab fragments, and BAFF up-regulated the expression of Fc receptors (FcγR) on the surface of microglia, consequently, promote IgG binding to microglia.

**Conclusion:**

Our results suggest that lupus serum IgG causes inflammatory responses of microglia by involving the Fc signaling pathway and the activity could be up-regulated by BAFF. Accordingly, disruption of the FcγR-mediated signaling pathway and blockade of microglia activation may be a therapeutic target in patients with neuropsychiatric lupus erythematosus.

## Background

Systemic lupus erythematosus (SLE) is an autoimmune disease characterized by high levels of autoantibody and damages in multiple organs, including central nervous system [1, 2]. Neuropsychiatric lupus erythematosus (NPSLE) has been reported as a common syndrome of SLE [3, 4]. It is widely accepted that tissue damage in SLE is associated with autoantibody production and immune complex formation and deposition [5]. Previous studies on NPSLE have reported some antibodies involving in the neural damage in the central nervous system (CNS). For example, anti-dsDNA antibody, recognizing a subunit of the *N*-methyl-d-aspartate receptor (NMDAR) in neurons, was proposed to bind to neurons undergoing apoptosis and contribute to cognitive impairments [6–8]. Anti-phospholipid antibodies (aPLs) were known as a risk for cerebral and other thromboembolic episodes [9]. Anti-ribosomal P-protein (anti-P) antibodies were also associated with psychosis in some studies [10]. Although these observations indicate that autoantibodies may play an important role in the brain damage involved in SLE, they only explain a minor proportion of several phenomena observed in the NPSLE patients. This may be due to the large variety of the antibodies in different SLE patients. Therefore, there may be other operative mechanisms underlying the neuropathological changes of SLE.

CNS has evolved as an immune-privileged system in which immune activities are tightly regulated [11, 12]. Microglia, the resident immune cells in the brain, plays a critical role in the immunological regulation in CNS. Under homeostatic conditions, microglia perform continuous immune surveillance of the brain parenchyma, provide trophic support to the neurons, and clear endogenous damage or exogenous factors [13, 14]. In contrast, in the presence of immune stimuli, microglia change to different phenotypes initiating defense responses [15]. When activating to the M1 phenotype, microglia elaborate pro-inflammatory cytokines and chemokines promoting inflammation and cytotoxic responses. In contrast, when adopting the M2 phenotype, microglia secrete anti-inflammatory gene products and trophic factors that promote repair, regeneration, and restore homeostasis [16, 17]. Unbalance between the M1 and M2 activation has been demonstrated to be an important pathological factor in many neuropsychological diseases, such as Alzheimer’s disease, Parkinson’s disease, multiple sclerosis and stress [18–22]. Recently, we have found that stimulation of microglia cultures with serum collected from SLE patients could induce the M1 activation of microglia, indicated by obvious morphological changes, expression of M1 phenotypic markers (MHCII and CD86), and overproduction of pro-inflammatory cytokines [23]. We, thus, hypnotize that microglia might serve as an important interface mediating the immune responses in the brain of SLE patients.

Here, we report that obvious M1 activation of microglia developed in normal mice following intraventricular injection of serum from patients with SLE, and the microglia activation required the presence of IgG in lupus serum. Furthermore, IgG deleted Fc fragments failed to binding to microglia, and BAFF could enhance the binding of IgG to microglia by up-regulating the FcγR on the surface of microglia. The identified components of the lupus serum-induced neuroinflammation suggest a possibility to treat NPSLE by blocking FcγR/BAFF signaling.

## Methods

### Serum collection

A total of 28 hospital patients, who fulfilled the American College of Rheumatology (ACR) classification criteria for SLE [24] from November 2017 to March 2018, and 23 healthy age- and gender-matched subjects were enrolled in this study. Sera from the SLE patients and healthy controls were prepared by centrifugation at 3000 rpm for 10 min in a clinical centrifuge and then stored at − 80 °C prior to use. The study protocol was approved by the Ethics Committee of the First Affiliated Hospital of China Medical University.

### IgG and Fab fragment preparation

IgG was purified from SLE patients’ serum pools using the HiTrap Protein G columns (GE Healthcare, Freiburg, Germany). The samples were further concentrated with Amicon Ultra-15 centrifugal filters (Merck Millipore, Massachusetts, USA). Western blot analysis was used to confirm the purity of IgG.

IgG was divided into Fab and Fc fragments by enzymatic digestion. Pepsin (#P6887 Sigma-Aldrich, St. Louis, USA) was mixed with IgG at a ratio of 1:20. The solution was then maintained at 37 °C in a water bath for 6 h. Digestion was interrupted by adjusting the solution pH to 7.4 through the addition of 3 M Tris–HCl. Then, HiTrap Protein G HP columns (GE Healthcare, Freiburg, Germany) and Amicon Ultra-15 centrifugal filters (Merck Millipore, Massachusetts, USA) were used to obtain purified Fab segments.

### Mice

C57BL/6 males were purchased from Vital River Laboratory (Beijing, China) and were maintained in community cages under standard housing conditions. The mice were kept under a reversed 12:12 h dark/light cycle. The animal care and experimental procedures were performed according to institutional guidelines and approved by the Animal Care and Use Committee of China Medical University (No. KT2018060). All surgeries were performed under anesthesia, and all efforts were made to minimize animal suffering.

### Intraventricular injection

Male mice at 9 weeks of age were anesthetized with chloral hydrate (0.1 ml/10 g, 4%, i.p). The animal was placed in a stereotaxic frame (#DW-2000, Taimeng, Chengdu, China) under sterile conditions, and after an incision in the skin, the cranium was exposed. And small holes were drilled over the parietal bone to injection. A 24-gauge needle connected with polyethylene tubing to a 10-μl Harvard Apparatus syringe pump system (Pump 11 Elite) was inserted into lateral ventricle corresponding to stereotactic coordinates (AP = − 0.3 mm, ML = 1.0 mm, and DV = − 2.5 mm). Injection (2 µl) of serums or artificial cerebrospinal fluid (ACSF; glucose, 5 mM; CaCl_2_, 1 mM; NaCl, 125 mM; MgCl_2_, 1 mM; NaHCO_3_, 27 mM; KCl, 0.5 mM; Na_2_SO_4_, 0.5 mM; NaH_2_PO_4_, 0.5 mM; and Na_2_HPO_4_, 1.2 mM) was performed at the rate of 0.4 µl/min. The injection needle remained in place for at least 10 min after the infusion before being pulled out to prevent backflow of the injectate. The hole was then enclosed using dental cement and the incision was sutured. All animals were handled according to the approved animal protocol at the China Medical University.

### Brain histology

Male mice were sacrificed and extensively perfused with cold PBS. Brains were dissected into right and left hemispheres. The left hemisphere of the brain was fixed in 4% paraformaldehyde for 24 h at 4 °C. The right brain hemisphere was snap-frozen for RNA extraction. At least 24 h before sectioning, the fixed brains were transferred to a 20% sucrose (w/v) solution for cryoprotection. Coronal sections of 10 µm were cut on a freezing microtome, mounted on gelatin-coated slides.

### Isolation of microglia

Microglia were isolated from complete brains at 48 h after intraventricular injection. The mice were anesthetized by i.p. injection of 4% chloral hydrate and were transcardially perfused with pre-cooled PBS. Microglia isolation was performed according to Cardona et al. [25]. The brains were removed and rinsed in ice-cold phosphate buffer saline (PBS) and were placed on a 35 mm culture dish containing 3–5 ml of ice-cold PBS. The brain was cut into pieces of about 1 × 1 mm with scissors. All above of operation was performed on ice. The crushed brain fluid was transferred to a 15 ml centrifuge tube, and 5 ml of 0.25% trypsin was added. The mixture was digested in a 37 °C water bath for 20 min, and the centrifuge tube was manually shaken every 5 min. The mixture that had been completely digested was filtered through a 100 µm filter and centrifuged at 800*g* for 10 min. The obtained cell pellet was re-suspended in 10 ml of 37% percoll, then 10 ml of each of 30% and 70% percoll was gently added thereto by syringe, and centrifuged at 1100*g* for 30 min without acceleration and brake. After centrifugation, approximately 8 ml of a white hazy mononuclear cell layer was harvested from the interphase between the 37% and 70% percoll layers. The cells were washed with an equal amount of 1× PBS, and centrifuged at 1100*g* for 15 min. The cell pellets were dissolved in FACS buffer (PBS containing 1% bovine serum albumin [BSA; #V900933, Sigma-Aldrich, St. Louis, USA]) for flow cytometric analysis.

### Flow cytometry

We firstly checked the number of viable cells in single cell suspensions using trypan blue dye (#C0040, Solarbio, Peking, China). Cell suspension was mixed with 0.4% trypan blue in a ratio of 9:1 (final concentration 0.04%), dyed for 3 min and counted with the hemacytometer and binocular microscope. The cell viability was higher than 90%. Then, the following antibodies were used for mouse microglia surface staining: PE-Cy7 rat anti-mouse CD45 (#130-110-799, MiltenyiBiotec, BergischGladbach, Germany), APC-Cy7 rat anti-mouse CD11b (#130-109-366, MiltenyiBiotec, BergischGladbach, Germany), FITC rat anti-mouse MHCII (#11-5322-81, Invirogen, Carlsbad, USA), isotype for MHCII (#11-4031-81, Invirogen, Carlsbad, USA), Percp-cy5.5 rat anti-mouse CD206 (#141715, BioLegend, San Diego, USA) and isotype for CD206 (#400531, BioLegend, San Diego, USA). The antibodies were added to the FACS cell re-suspension in a ratio of 1:100. After staining, the cells were washed once, re-suspended in 300 µl of paraformaldehyde, and transferred to BD FACS tubes.

For the analysis of FcγR expression in cultured microglia, Fc blocks were added to avoid non-specific staining. Cells were calculated and 1 × 10^6^ cells were stained with anti-mouse immune cell surface markers for 15 min at 4 °C: FcγRI-PerCP/Cy5.5 (#139307, BioLegend, San Diego, USA), isotype for FcγRI-PerCP/Cy5.5 (#400149, BioLegend, San Diego, USA), FcγRIIB-APC (#17-0321-80, Invirogen, Carlsbad, USA), isotype for FcγRIIB-APC (#17-4724-41, Invirogen, Carlsbad, USA), FcγRIII-FITC (#101305, BioLegend, San Diego, USA) isotype for FcγRIII-FITC (#400505, BioLegend, San Diego, USA), FcγRIV-PE (#149503, BioLegend, San Diego, USA) and isotype for FcγRIV-PE (#400907, BioLegend, San Diego, USA). Each antibody was added to its corresponding isotype control to define the gating and exclude non-specific staining. The flowcytometry machine model is FACSAriaTMIIu (BD Biosciences, Franklin Lakes, USA) and the results were acquired with CellQuest software and then analyzed in FlowJo v10 software (Tree Star, Ashland, OR, USA).

### Microglial cell cultures

The mouse microglia cell line (BV-2 microglia) was originally obtained from the Cell Resource Centre (Peking Union Medical College). The cells were cultured in 75-cm^2^ flasks with Dulbecco’s Modified Eagle Medium (DMEM)/high glucose supplemented with 10% fetal bovine serum (FBS), 100 units/ml of penicillin and 100 μg/ml of streptomycin and maintained in a 5% CO_2_ incubator at 37 °C. When the cells reached 80% confluence, they were sub-cultured by replacing the culture medium and the adherent cells were aspirated with a scraper, and then seeded into 96-well (3–8 × 10^4^ cells/well) or 6-well (1 × 10^6^ cells/well) plates. Twenty-four hours later, BV-2 microglia were used for the experiments.

### Immunofluorescence staining

For staining of brain section, the sections were first blocked with 10% blocking serum in PBS and then incubated with the indicated primary antibodies Iba-1 (1:100 dilution in 1× PBS, #10904-1-AP, Proteintech, Chicago, USA) overnight at 4 °C. Slides were then incubated with secondary antibody for 2 h at room temperature. Goat anti-rabbit IgG(H + L)-594 (1:300 dilution in 1% BSA, #SA00006-4, Proteintech, Chicago, USA) was used to detect Iba-1.

For staining of cultured cells, BV-2 microglia, plated 24 h on poly l-lysine/laminin glass coverslips (Sigma-Aldrich, St. Louis, USA), were fixed with 4% (*v*/*v*) paraformaldehyde in 1× PBS at room temperature for 30 min and washed with 1× PBS for 3 times, permeabilized with 0.1% (*v*/*v*) Triton X-100 in 1× PBS at room temperature for 20 min and washed with 1× PBS for 3 times afterwards. They were subsequently blocked with 1× PBS containing 5% BSA at room temperature for 1 h. Primary antibody against ionized calcium binding adaptor molecule (Iba-1) (1:100 dilution in 1× PBS, #10904-1-AP, Proteintech, Chicago, USA) was incubated at room temperature for 2 h. Subsequently, cells were incubated with goat anti-human IgG(H + L)-FITC (1:200 dilution in 1% BSA, #SA00003-12, Proteintech, Chicago, USA) and goat anti-rabbit IgG(H + L)-594 (1:300 dilution in 1% BSA, #SA00006-4, Proteintech, Chicago, USA) at room temperature for 1 h in dark. After washing with 1× PBS for 3 times, cell nuclei were stained with DAPI at room temperature for additional 5 min in dark. Washing steps following DAPI staining were three times with 1× PBS containing 0.05% Tween-20. Cover slips were finally mounted with fluorescence decay resistance sealing reagent.

### Cytokine assay in brain tissues

The mice were deeply anesthetized with 4% chloral hydrate, and the brains were removed rapidly. Brain tissues from both hemispheres were mixed and homogenized on ice in 0.01 M PBS (pH, 7.4) and centrifuged at 12,000 rpm for 15 min at 4 °C to remove cell debris. The supernatants were collected and stored in aliquots at − 80 °C until the measurement. Interleukin (IL)-1β (#EK0394), tumor necrosis factor (TNF)-α (#EK0527), IL-4 (#EK0405), IL-6 (#EK0411) and IL-10 (#EK0417) were measured in the supernatants by enzyme-linked immunosorbent assay (ELISA) according to the manufacturer’s instructions (Boster, Wuhan, China). The OD was determined at 450 nm in the Bio-Rad Model 550 Microplate Reader (Bio-Rad, Hercules, CA, USA) and the corresponding mouse recombinant protein was used as standard. All treatments were completed at least 3 times, and the data were expressed as the mean pg/ml ± SEM.

### Quantitative PCR

Total RNA was extracted from cultured BV-2 microglia using Axyprep™ Multisource Total RNA Miniprep Kit (#AP-MN-MS-RNA-50, Axygen, Silicon Valley, USA). cDNA was synthesized using Primescript™ RT reagent Kit with gDNA Eraser (#RR047A, TaKaRa, Dalian, China). qPCR was performed with TB Green™ Premix EX Taq™ II (#RR820A, TaKaRa, Dalian, China) and each forward and reverse primer. The qPCR conditions were: 1 denaturation cycle at 95 °C for 30 s; 36 amplification cycles of 95 °C for 10 s, 60 °C annealing for 30 s, and elongation at 72 °C for 15 s; followed by 1 dissociation cycle (Mx3000 P QPCR System, Stratagene, now Agilent Technologies, La Jolla, CA). The relative expression levels were quantified using the 2^−∆∆Ct^ method and were normalized to the measurement for GAPDH. Primers were designed using Primer Express software and purchased from Sangon Biotech. The sequence of primer is

- FcγRI: forward 5′-GCGGAAAGAGAAGATGCTGGATTC-3′, reverse 5′-CTTCTCTCTCTGCAGCCTGTGTAT-3′;
- FcγRIIB: forward 5′-GAAACCATCACGCTAAGGTGCC-3′, reverse 5′-TGGTGCAGTGTCCTTCCTAGAC-3′;
- FcγRIII: forward 5′-GGTACCACACTGCTTTCTCCCT-3′, reverse 5′-ACTTCCTCCAGTAATCCCTCGG-3′;
- FcγRIV:forward 5′-CCACCGTGGCATCAAATCACAT-3′, reverse 5′-GTCCTGAGGTTCCTTGCTCCAT-3′;
- GAPDH: forward 5′-CATGGCCTTCCGTGTTCCTA-3′, reverse 5′-ATGCCTGCTTCACCACCTTCT-3′.

### Statistical analysis

Statistical analysis was performed with IBM SPSS statistics software. All data were expressed as the group mean ± SEM unless otherwise stated. All experiments were performed at least six times, and continuous values among groups were analyzed using one-way ANOVA with Tukey’s post hoc test (for normally distributed data). The differences between two groups were calculated by a two-tailed unpaired t-test. The results were considered significant at p < 0.05.

## Results

### Intraventricular injection of SLE-serum induced M1-polarized microglia activation

**Fig. 1:**
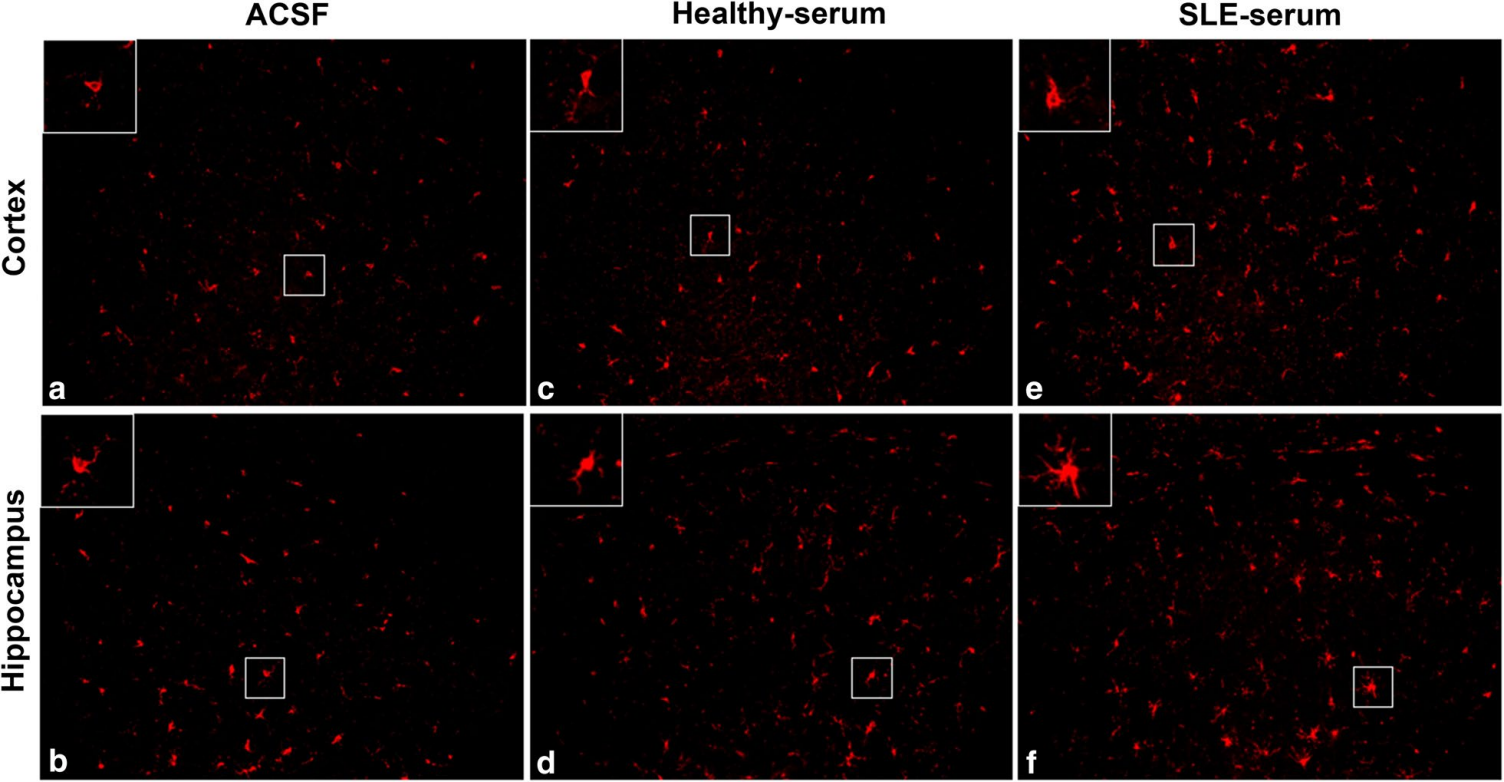
Microglia activation following SLE-serum, healthy-serum or ACSF injection. Microscopic images of the Iba-1 immunolabelled cortical (**a**, **c** and **e**) and hippocampal (**b**, **d** and **f**) sections depicting morphological transformation of microglia after injection of ACSF (**a** and **b**), healthy-serum (**c** and **d**) and SLE-serum (e and f). The upper-left square represents the amplification of the middle square i.e. from resting state in ACSF and healthy-serum injection to activated phenotype in SLE-serum injection

**Fig. 2:**
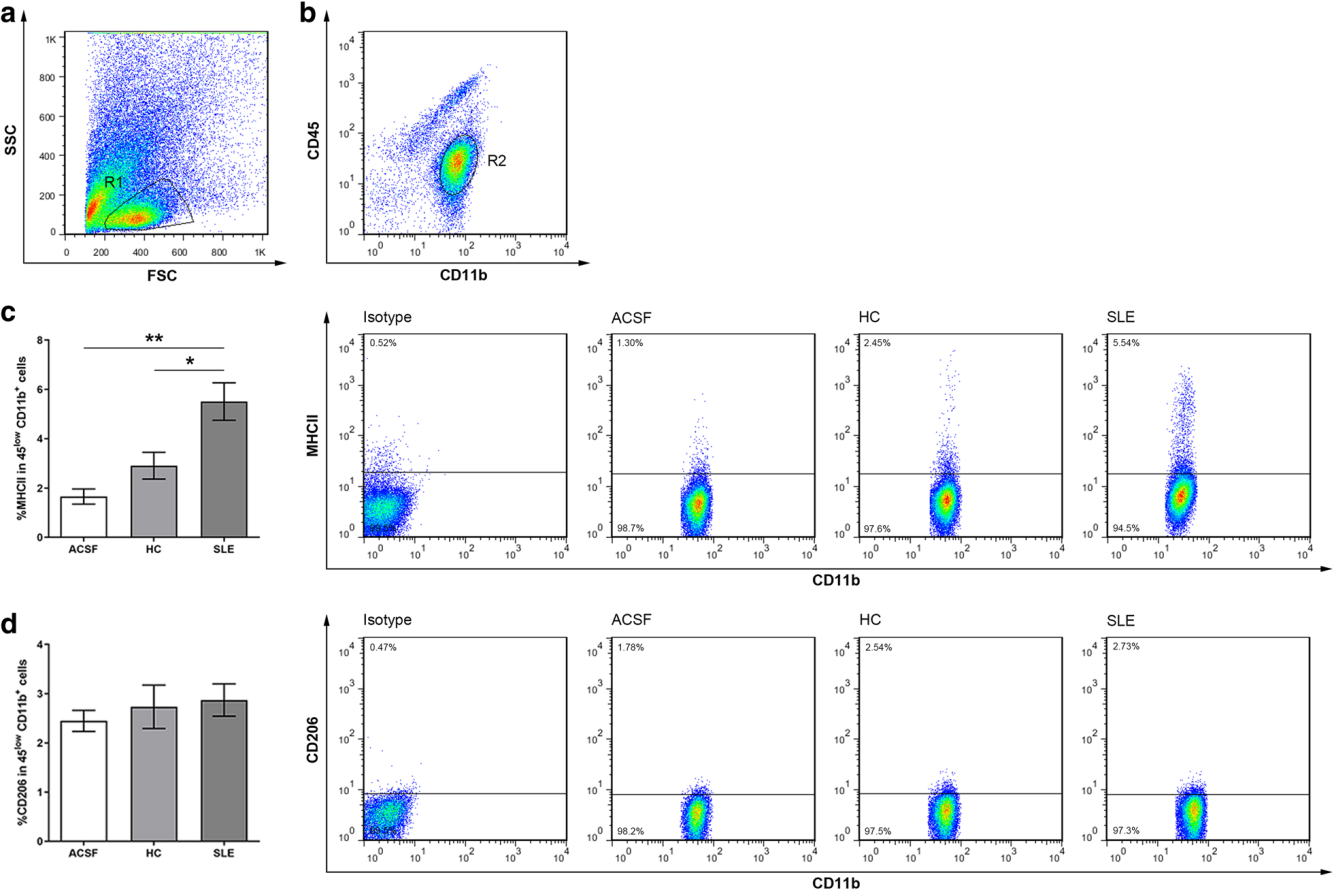
Flow cytometric analysis of microglia surface markers in SLE-serum, healthy-serum and ACSF-treated mice (n = 16, 14 and 10). **a** Cells were defined by gate R1 to eliminate unwanted events, such as cell debris. **b** Microglias were identified by their CD45^low^CD11b + phenotype (R2). **c** Isotype for MHCII was used to define the gating of M1 cells. In this population, SLE-serum led to a significantly increased percentage of MHC-II + CD45^low^CD11b^+^ cells. **d** Isotype for CD206 was used to define the gating of M2 cells. In contrast, percentage of CD206^+^ was not significantly changed by SLE-serum treatment in the CD45^low^CD11b^+^ cells. Bars represent the mean ± SEM of mice groups received injection of SLE-serum, healthy-serum or ACSF. Pictures of c and d show representative histogram and flow dot plots of MHC-II and CD206 expression, respectively, in the CD45^low^CD11b^+^ population. *p < 0.05; **p < 0.01

To examine whether microglia were activated by intraventricular injection of SLE-serum, we performed immunohistological staining of Iba-1 (a microglial marker) on the brain slices of the cortex and hippocampus at 48 h after SLE-serum, healthy-serum, or ACSF (vehicle control) injection. Iba-1 protein expressed on the cell surface is prevalently used to study the activated states [26, 27] and morphological changes in the microglia [28, 29]. In the mice received ACSF and healthy-serum injection, Iba-1 + microglia showed a resting morphology, indicated by ramified wispy appearance with round lightly stained cell body (Fig. 1a–d). In contrast, injection of SLE sera induced a phenotypic transformation from ramified to activated states bearing retracted, thick processes and large irregular cell body (Fig. 1e, f). Using the other set of mice, we isolated microglia from complete brains at 48 h after intraventricular injection, and used flow cytometry to analyze the expression of cell surface markers. The microglia were identified by their characteristic CD45^low^CD11b^+^ phenotype (Fig. 2a, b). In this population, we analyzed the expression of MHC-II and CD206, as the surface markers of M1 and M2 activation, respectively. Comparing to ACSF treatment, both SLE and healthy-serum treatment resulted in an increase of MHC-II (M1 surface marker) expression in the CD45^low^CD11b^+^ cells, but SLE-serum induced more severe M1 activation of microglia than did healthy-serum (Fig. 2c). In contrast, there was no significant difference between the M2-associated parameter CD206 expressions after different treatments (Fig. 2d).

**Fig. 3:**
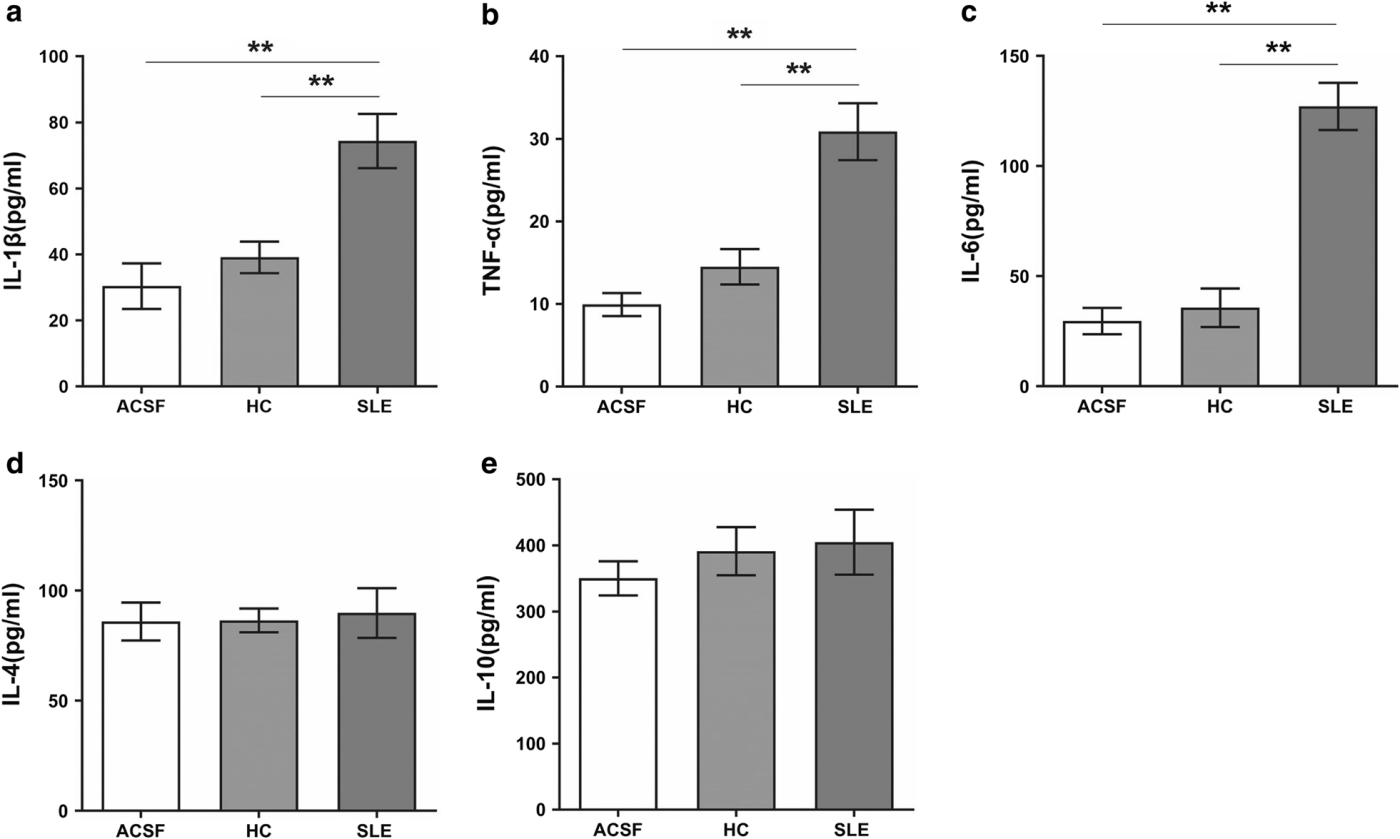
Cytokine expression levels in the brains measured 48 h after injection of SLE-serum, healthy-serum or ACSF. IL-1β (**a**), TNF-α (**b**), IL-6 (**c**), IL-4 (**d**) and IL-10 (**e**) expression in brains measured 48 h under different conditions. Data are presented as mean ± SEM with n = 6 mice per group. **p < 0.01

Consistently, ELISA assays revealed that the level of pro-inflammatory cytokines (IL-1β, TNF-α and IL-6) in the brain tissues were elevated after SLE-serum treatment (Fig. 3a–c), while the production of anti-inflammatory cytokines (IL-4 and IL-10) remained unchanged (Fig. 3d, e).

### IgG is a major contributor to the microglia activation induced by SLE-serum

**Fig. 4:**
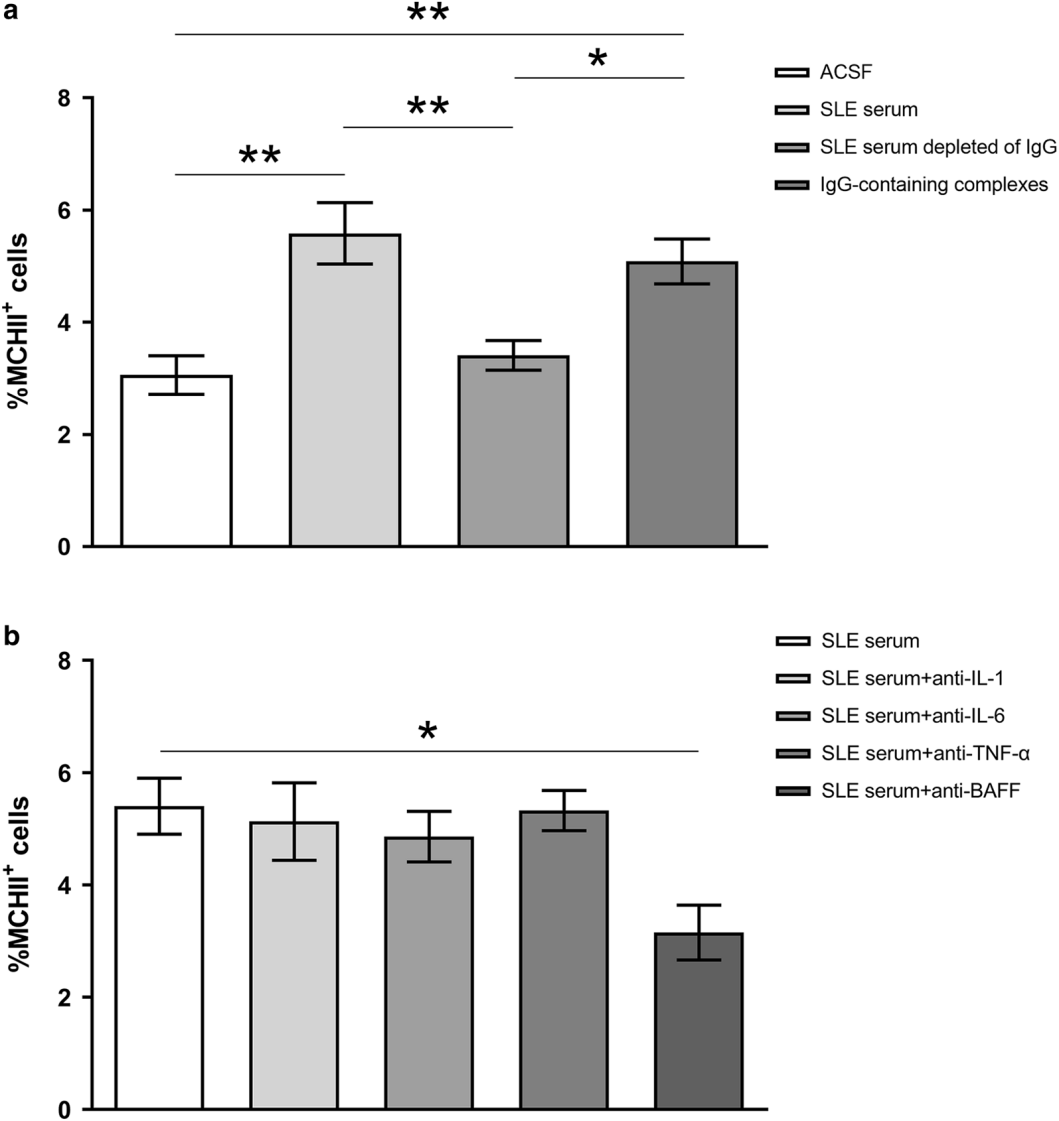
Percentages of MHCII^+^ microglia under different conditions. **a** Effect of IgG in the SLE serum on the microglia activation. Bars represent the mean ± SEM of percentages of MHCII^+^ microglia in the mice of different group. **b** Effect of cytokine neutralization on the increase of percentages of MHCII^+^ microglia induced by SLE serum. *p < 0.05; **p < 0.01

To determine whether IgG present in SLE sera is a major contributor to SLE serum-induced microglia activation, we used affinity chromatography to remove IgG from SLE sera. IgG depletion was confirmed by electrophoresis. We found that the increase of percentage of MHC-II^+^ microglia was largely abrogated in mice injected with SLE sera depleted of IgG. But when we injected IgG-containing complexes isolated from protein G agarose beads in mice, an increase of MHC-II^+^ microglia were observed (Fig. 4a). These data indicate that IgG are required for SLE serum-induced M1 activation of microglia.

### BAFF in the SLE-serum contributes to microglia activation

We further examined whether the cytokines in SLE sera contribute to the microglia activation. We used exogenous monoclonal antibodies to neutralize IL-1, IL-6, TNF-α or BAFF in the sera, respectively, and then examined whether SLE-serum induced increase of MHC-II expression is suppressed. As shown in Fig. 4b, the SLE sera pre-treated withanti-IL-1 (#AF-201, R&D, Minnesota, USA), anti-IL-6or anti-TNF-α (#10395-R508, #10602-MM0N1, Sino Biological, Beijing, China) induced similar percentage of MHC-II expression in microglia as did non-pretreated SLE sera. However, the SLE sera pre-treated with anti-BAFF antibodies (#MAB124, R&D, Minnesota, USA) induced significantly lower percentage of MHC-II expression than the non-pretreated SLE sera. These results suggest that BAFF may facilitate the IgG in SLE-serum to activated microglia.

### IgG from SLE-serum binds to microglia through Fc fragment

**Fig. 5:**
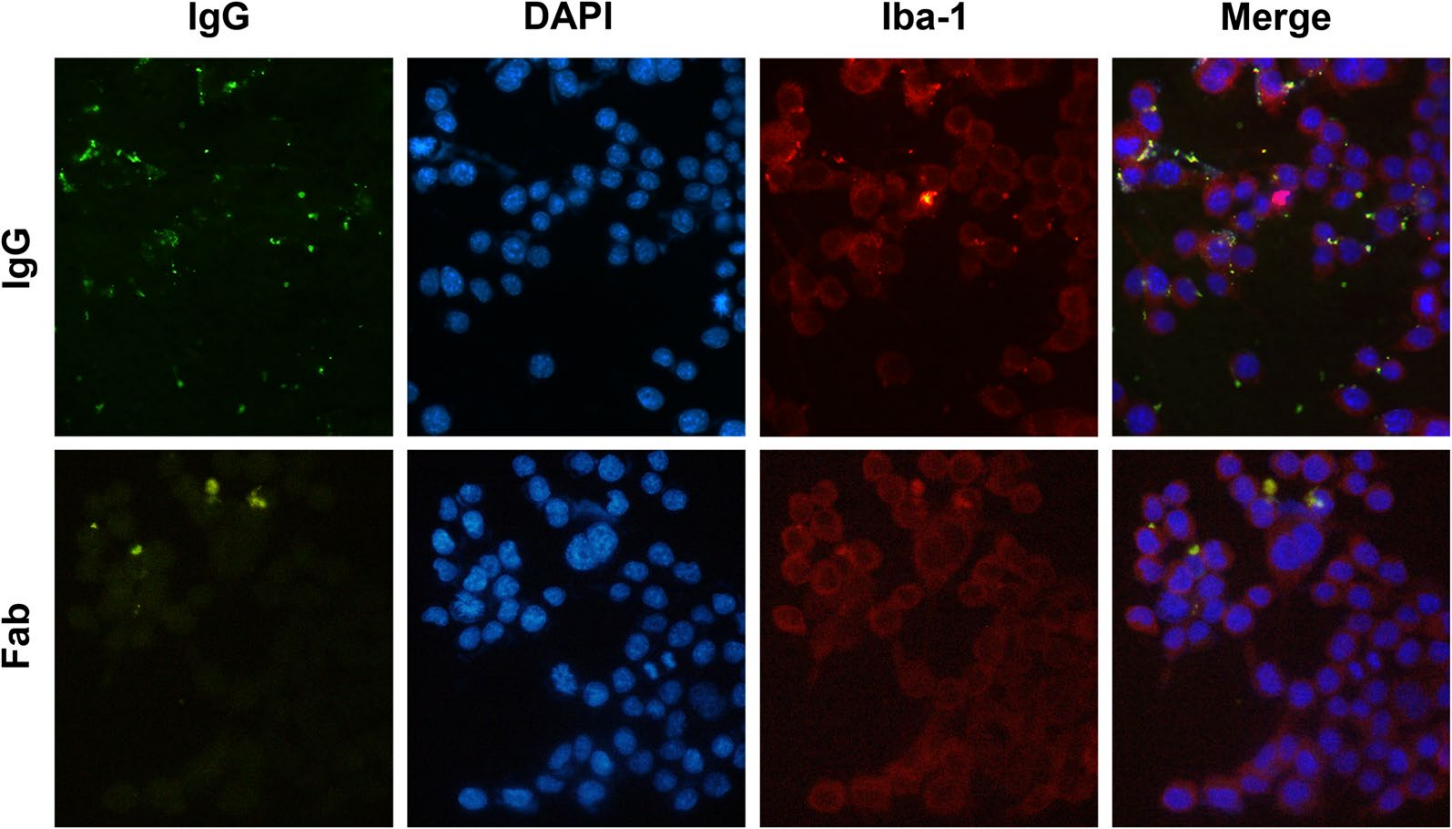
Immunofluorescence staining of BV-2 microglia incubated with SLE-IgG or Fab fragment of SLE-IgG. IgG is stained by FITC (green). DAPI is a marker of cell nuclei (blue). Iba-1 is a marker of microglia (red)

We further used in vitro microglia cell cultures (BV-2 microglia) to examine whether the IgG of SLE-serum interacts with microglia. BV-2 microglia is cell line of murine origin immortalised with v-raf/v-myc oncogenes and commonly used in microglia studies. The BV-2 microglias are similar in morphology to isolated microglias, express inflammatory mediators and display phagocytic activity [30]. Immunofluorescence staining showed that IgG bound to the surface of microglia after incubating the microglia with IgG purified from SLE sera (Fig. 5). IgG is composed of Fab and Fc fragments. Fab fragment specifically contacts with the antigens, while Fc fragment non-specifically interacts with FcγR in the immunological effector cells resulting in phagocytosis and/or releasing inflammatory mediators [31]. Therefore, there are two possible mechanisms for the binding of IgG to microglia. One is that microglia may contain some antigens, which can be recognized by the Fab domain of IgG. The other is that Fc fragment of IgG may bind to the FcγR expressed on the surface of microglia [32–34]. To testify these possibilities, we incubated the microglia with Fab fragments prepared from IgG of SLE patients, and found that Fab fragments failed to bind to microglia (Fig. 5). Thus, the binding of IgG to microglia is Fc dependent.

### BAFF enhances the binding of IgG to microglia in BV-2

**Fig. 6:**
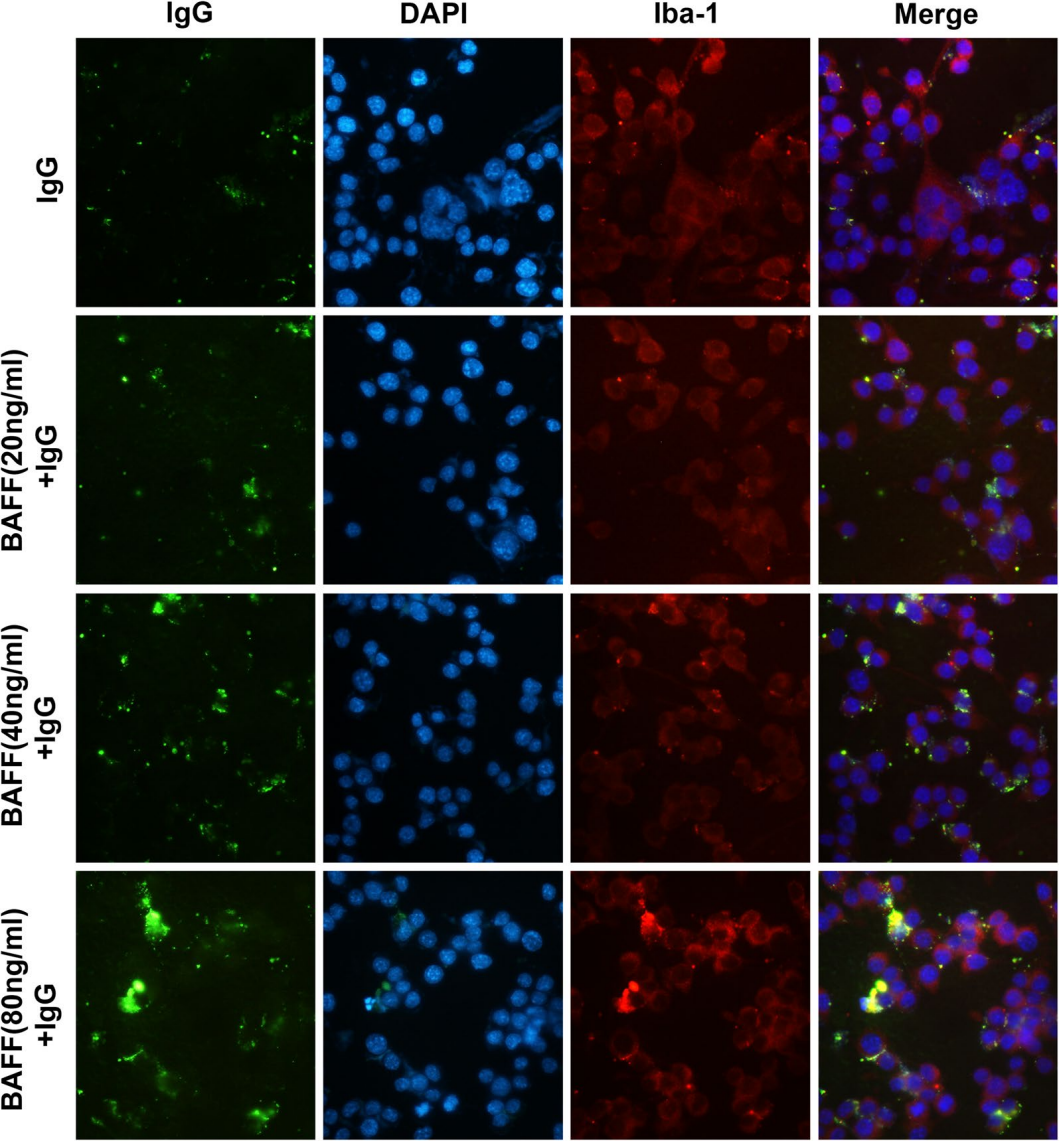
Immunofluorescence staining of BV-2 microglia incubated with SLE-IgG combined with different doses of BAFF. IgG is stained by FITC (green). DAPI is a marker of cell nuclei (blue). Iba-1 is a marker of microglia (red)

Next, we examined whether BAFF affects the binding of IgG to microglia. We incubated the microglia with SLE-IgG together with mouse recombinant BAFF (#8876-BF, R&D, Minnesota, USA). The results indicate that BAFF enhanced the binding of IgG to microglia in a dose dependent manner (Fig. 6).

### BAFF up-regulates the expression of FcγRs in microglia

**Fig. 7:**
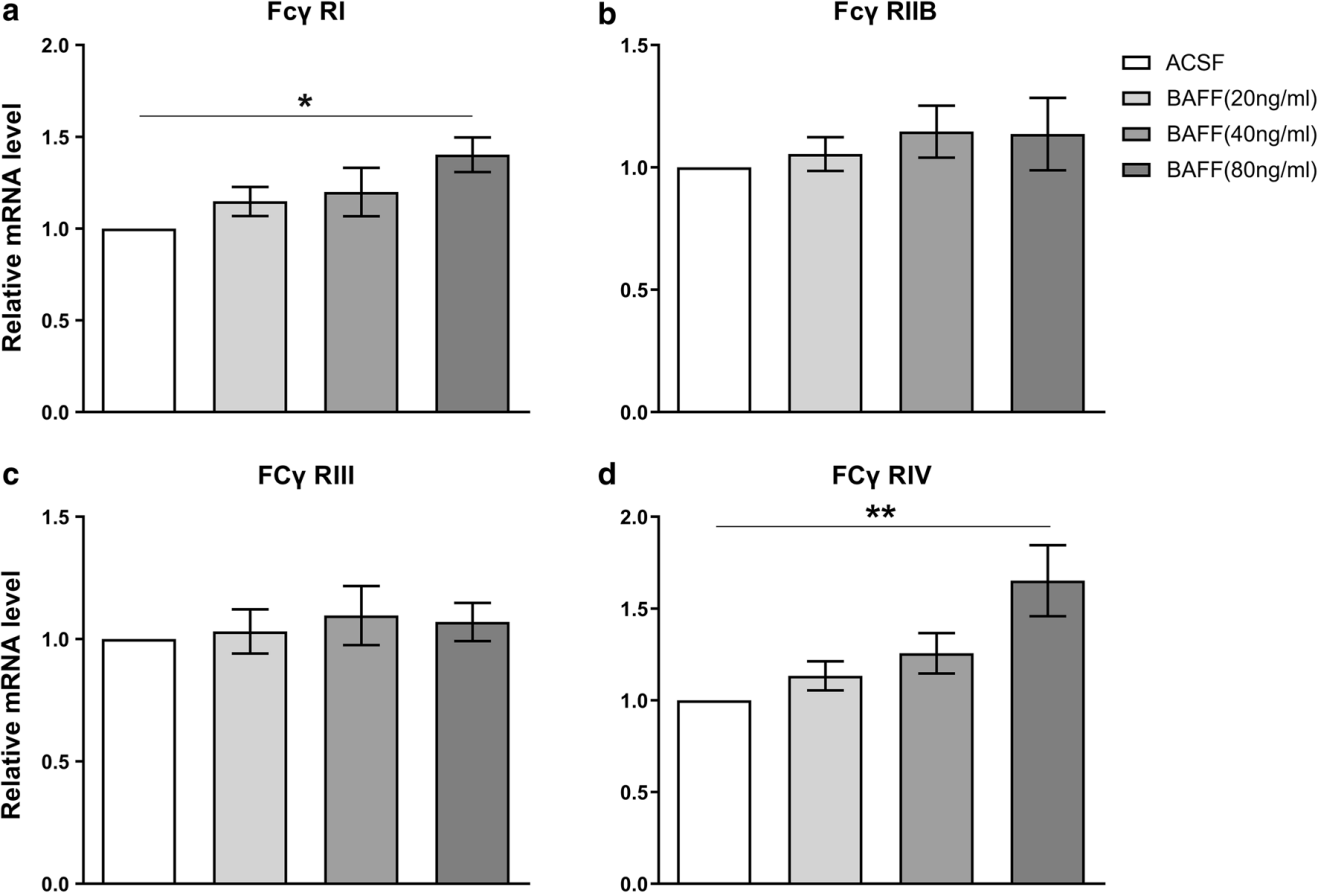
The mRNA expression level of FcγRs in BV-2 microglia following stimulation with BAFF. The mRNA level of FcγR I (**a**), FcγR IIB (**b**), FcγR III (**c**) and FcγR IV (**d**) in BV-2 microglia. Bars represent the mean ± SEM of mRNA level in the microglia incubated with different doses of BAFF. *p < 0.05; **p < 0.01

**Fig. 8:**
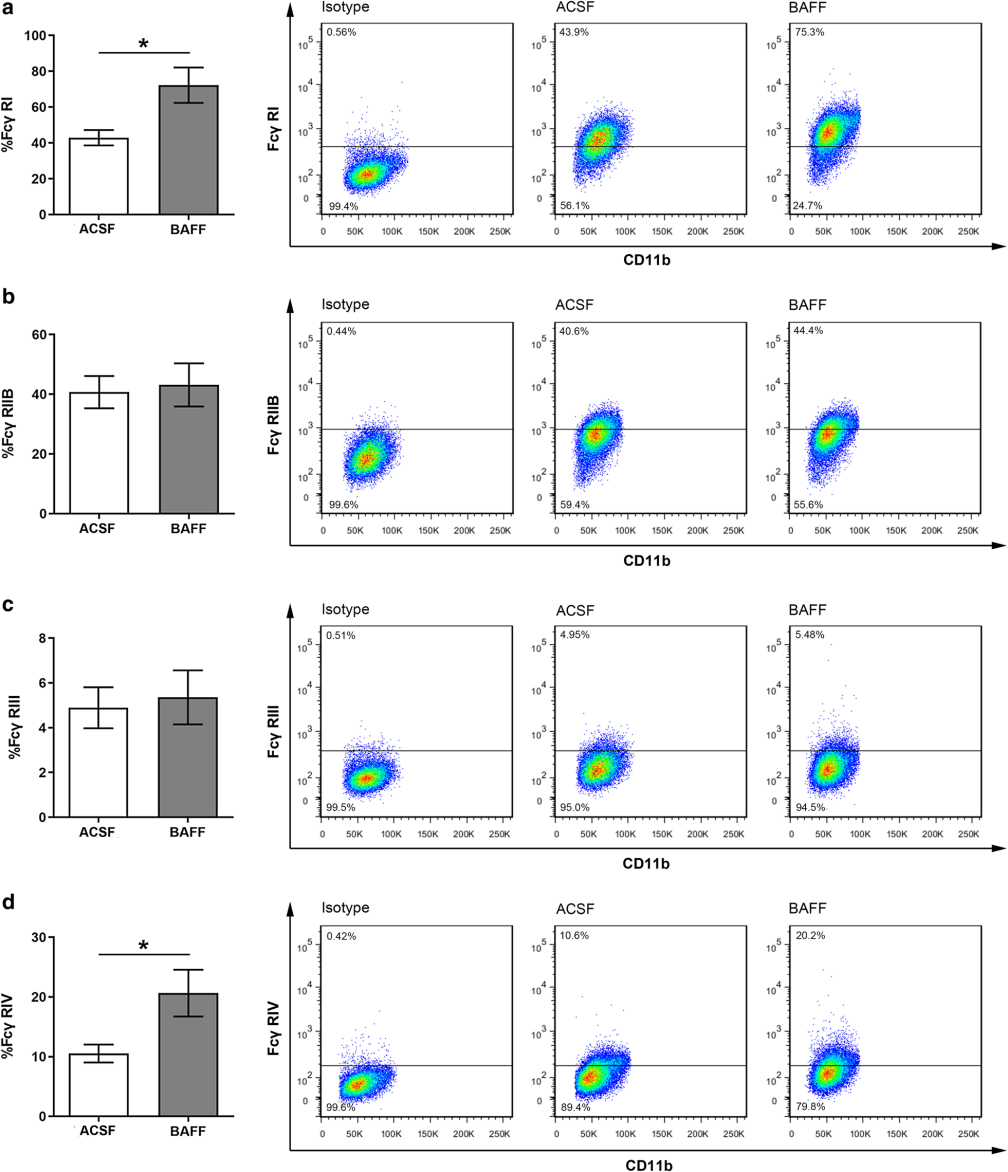
Flow cytometric analysis of FcγRs expression on microglia surface, stimulated by ACSF or 80 ng/ml BAFF. FcγR I (**a**), FcγR IIB (**b**), FcγR III (**c**) and FcγR IV (**d**) expression under different conditions. Bars represent the mean ± SEM of FcγRs expression. *p < 0.05

Finally, we asked whether BAFF can facilitate the binding of IgG and microglia by up-regulating the expression of FcγRs in microglia. We assessed FcγR expression levels of microglia stimulated with BAFF. FcγRs have four main subtypes: FcγRI, FcγRIIB, FcγRIII and FcγRIV [35]. Analysis using qPCR demonstrated that mRNA expression of FcγRI and FcγRIV was enhanced following stimulation with BAFF (Fig. 7a, d). In contrast, we did not observe any significant differences in the mRNA expression of FcRIIB and FcγRIII (Fig. 7b, c). We further examined the protein expression of FcγRs in the microglia surface using flow cytometry, and observed a corresponding increased expression of FcγRI and FcγRIV, following stimulation with 80 ng/ml BAFF (Fig. 8).

## Discussion

In this study, we have demonstrated that intraventricular injection of SLE-serum results in M1 activation of microglia in CNS and IgG in the sera plays a key role in the activation of microglia. BAFF is also involved in the process of microglia activation. We further found that the binding of IgG to microglia was dependent on the Fc fragment, and BAFF can facilitate this process by up-regulating the expression of FcγRs on microglia.

Previously, several autoantibodies acting upon neurons have been connected to distinct pathologies in NPSLE patients, such as anti-ribosome P [10] and anti-NMDAR antibodies [6–8] discussed above. Because the presence of a large diversity of autoantibodies in SLE, we cannot identify a single pathologic antibody response for NPSLE. On the other hand, regardless of their specificity, all the antibodies may evoke microglia activation which mediates a common inflammatory response in the brain.

Upon inflammatory stimulation, microglia can be polarized into M1 and M2 phenotypes [36]. The M1 phenotype, as marked by the up-expression of MHC-II is optimized to facilitate the elimination of intracellular pathogens through the release of Th1 cytokines such as IL-1β and TNF-α [37]. Th2 cytokines such as IL-4 and IL-10, on the other hand, are generally produced in response to M2 polarization and may provide a protective mechanism to prevent hyper immune responses and bystander brain damage [38]. In this study, we assessed the polarization of microglia by flow cytometry. We isolated microglia of mice from complete brains using mechanical dissociation and density gradient centrifugation and avoiding enzymatic digestion, adapted from the method described by Campanella et al. [39]. The microglia population was identified by its surface expression of CD11b and low or intermediate expression of CD45. Brain macrophages (present in the perivascular regions, choroidal plexus, and meninges) are also positive for CD11b but show high expression of CD45 [40]. Blood macrophages were excluded by transcardiac perfusion with PBS. Our results showed that the expression of MHC-II on microglia was increased by treatment of SLE IgG, suggesting a M1 polarization of microglia. Consequently, the concentrations of pre-inflammatory cytokines (IL-1β, TNF-α and IL-6) were elevated in the brain tissues.

It has been well demonstrated that signaling events initiated by IgG interacting with FcγR on monocytes and macrophages trigger internalization of the FcγR-IgG-antigen complex and ultimately result in phagocytosis and/or release of inflammatory or cytotoxic mediators [41, 42]. These responses are well described in peripheral tissues, using the (reversed) Arthus reaction, a generally accepted experimental model of antibody-mediated inflammation [43]. The expression of FcγR has been found in microglia [44], and activation of FcγRs in microglia has been implicated in the pathologies of some CNS disorders [44–46]. Consistent with previous observations, we found FcγR express in microglia, and Fab portion of IgG, lack Fc portion, failed to bind to microglia. Thus, we propose that IgG of SLE serum may activate microglia through Fc/FcγR interaction.

FcγRs can be classified into four types: FcγRI, FcγRIIB, FcγRIII, and FcγRIV [35]. FcγRI, FcγRIII, and FcγRIV are activating receptors, while FcγRIIB is inhibitory receptor. In the healthy brain, microglias express all the four types of FcγRs at a low level [44]. The expression level can be up-regulated in response to a number of different insults to the CNS, such as treated with IFN-γ, TNF-a, and lipopolysaccharide (LPS) in vitro [47, 48], during acute phase of infection [49] or at multiple sclerosis lesions [50, 51]. Here, we found that BAFF significantly increased the expression of activating FcγR (FcγRI and FcγRIV), but had no effect on the expression of inhibitory FcγRIIB. This can explain the results that BAFF enhanced the binding of IgG and microglia, and neutralizing BAFF suppressed the microglia activation induced by SLE serum. BAFF is a member of the TNF family and is expressed by many immunological cells including monocytes, dendritic cells, neutrophils, activated T cells and malignant B cells [52–54]. Originally, BAFF is known as a vital contributor to the survival of B lymphocytes and maintenance of ensuing effective humoral immune responses [55–57]. In the population of SLE patients, elevated levels of soluble BAFF can be detected [58, 59] in serum. Mice genetically modified to over-express BAFF develop symptoms of a SLE-like autoimmunity [60, 61]. Recently BAFF level was found to be increased in the circulation and cerebral spinal fluid (CSF) of NPSLE patients [62]. It has been shown that microglias express the receptors of BAFF [63], suggesting that microglias are effector cells of BAFF. However, our results show that directly injection of SLE serum depleted IgG could not drive microglia polarization, indicating that the amount of BAFF in the sera was not high enough to activate microglia directly, which mainly play a facilitating effect on IgG induced microglia activation.

In the presence of an intact blood–brain-barrier (BBB), IgG is only present in the healthy brain at very low levels relative to plasma levels [64]. IgG is continuously removed from the CNS by an efficient process of reverse transcytosis across the BBB [65], mediated by the neonatal transport receptor [66, 67]. However, this may be altered under the condition of SLE, which associates with changes in BBB integrity. Accumulated IgG in the CNS can activate microglia as discussed above.

Our present results suggest that blockade of FcγRs in microglia may be a potential strategy to rescue NPSLE. It has been reported that intravenous immune globulin (IVIG) can block FcγRs [68], and is an effective therapy in a variety of autoimmune and chronic inflammatory diseases, including SLE [69–71]. IVIG can also increase expression of inhibitory receptor for IgG, FcγRIIB, which mediates the anti-inflammatory activity of IVIG [72]. Furthermore, BAFF production can be suppressed by IVIG treatment in vitro and in chronic-inflammatory demyelinating polyneuropathy (CIDP) patients [73]. Thus, our study provided experimental evidence supporting the feasibility of IVIG used in the treatment of NPSLE.

## Conclusions

We, for the first time, found that BAFF is involved in the interaction between SLE IgG and microglia through up-regulating the express of FcγRs. A better understanding of FcγRs function and crosslink between FcγRs and BAFF in the brain microenvironment will likely lead to a potential immunotherapy for NPSLE.

## Data Availability

The datasets used and analyzed during the current study are available fromthe corresponding author on reasonable request.

## Abbreviations

SLE: systemic lupus erythematosus
IgG: immunoglobulin G
qPCR: quantitative PCR
BAFF: B-cell activating factor
FcγR: Fc receptors
NPSLE: neuropsychiatric lupus erythematosus
CNS: central nerve system
NMDAR: *N*-methyl-d-aspartate receptor
aPLs: anti-phospholipid antibodies
anti-P: anti-ribosomal P-protein
ACSF: artificial cerebrospinal fluid
PBS: phosphate buffer saline
BSA: bovine serum albumin
IL: interleukin
TNF: tumor necrosis factor
ELISA: enzyme-linked immunosorbent assay
Iba-1: ionized calcium binding adaptor molecule
LPS: lipopolysaccharide
CSF: cerebral spinal fluid
BBB: blood–brain-barrier
